# HIV Preexposure Prophylaxis, by Race and Ethnicity — United States, 2014–2016

**DOI:** 10.15585/mmwr.mm6741a3

**Published:** 2018-10-19

**Authors:** Ya-lin A. Huang, Weiming Zhu, Dawn K. Smith, Norma Harris, Karen W. Hoover

**Affiliations:** ^1^Division of HIV/AIDS Prevention, National Center for HIV/AIDS, Viral Hepatitis, STD, and TB Prevention, CDC; ^2^Prosphere Tek, Inc., Alexandria, Virginia.

Preexposure prophylaxis (PrEP) with a daily, oral pill containing antiretroviral drugs is highly effective in preventing acquisition of human immunodeficiency virus (HIV) infection (*1*–*4*). The combination of tenofovir disoproxil fumarate (TDF) and emtricitabine (FTC) is the only medication approved by the Food and Drug Administration (FDA) for PrEP. PrEP is indicated for men and women with sexual or injection drug use behaviors that increase their risk for acquiring HIV (*5*). CDC analyzed 2014–2016 data from the IQVIA Real World Data — Longitudinal Prescriptions (IQVIA database) to estimate the number of persons prescribed PrEP (users) in the United States and to describe their demographic characteristics, including sex and race/ethnicity. From 2014 to 2016, the annual number of PrEP users aged ≥16 years increased by 470%, from 13,748 to 78,360. In 2016, among 32,853 (41.9%) PrEP users for whom race/ethnicity data were available, 68.7% were white, 11.2% were African American or black (black), 13.1% were Hispanic, and 4.5% were Asian. Approximately 7% of the estimated 1.1 million persons who had indications for PrEP were prescribed PrEP in 2016, including 2.1% of women with PrEP indications (*6*). Although black men and women accounted for approximately 40% of persons with PrEP indications (*6*), this study found that nearly six times as many white men and women were prescribed PrEP as were black men and women. The findings of this study highlight gaps in effective PrEP implementation efforts in the United States.

In 2012, FDA approved TDF/FTC for use as PrEP (*7*), and CDC published clinical practice guidelines for use of PrEP (*5*). A previous study estimated PrEP uptake among U.S. commercially insured populations and found that PrEP use increased among men during 2010–2014, but was very low among women (*8*). It is important to monitor PrEP uptake both among persons with private and public insurance. Because racial and ethnic disparities in HIV diagnoses exist in the United States (*9*), it is also important to better understand PrEP use by race/ethnicity. Monitoring trends in PrEP use can inform the development of interventions to ensure that PrEP is provided for persons who need it most to reduce racial and ethnic disparities in PrEP use and new HIV infections.

Data on antiretroviral drug prescriptions dispensed during 2014–2016 were extracted from the IQVIA database,* which captured prescriptions from all payers and represented approximately 92% of all prescriptions dispensed from retail pharmacies and 60%–86% dispensed from mail order outlets in the United States. The database included antiretroviral drugs dispensed, demographic variables of persons to whom the drugs were dispensed, and medical claims for these persons. IQVIA acquired medical claims and race/ethnicity data from various sources, including ambulatory, hospital, and consumer databases, and linked these data to persons in the prescription database. Among persons with any antiretroviral drug prescription (1,418,621), approximately 69% had medical claims data available, and race/ethnicity information was available for about 32%. CDC estimated the annual number of PrEP users based on a previously developed algorithm that discerns whether TDF/FTC was prescribed for PrEP or for HIV treatment, hepatitis B treatment, or HIV postexposure prophylaxis (*8*). For each year of the study, records of persons aged ≥16 years who had at least one TDF/FTC prescription were selected. Persons were then excluded if they had any diagnostic codes for HIV or hepatitis B infection that preceded their initial TDF/FTC prescription. In addition, persons prescribed TDF/FTC for ≤30 days were defined as postexposure prophylaxis users and excluded; the remaining persons with TDF/FTC prescribed for >30 days were considered PrEP users. Postexposure prophylaxis is recommended for 28 days; however, it is often prescribed for 30 days. The 30-day definition of postexposure prophylaxis was chosen to produce conservative estimates of TDF/FTC for PrEP. PrEP use among persons prescribed TDF/FTC for >28 days was also estimated, to assess the impact of different duration of drug use on the estimates. PrEP use estimates were reported by age group, sex, geographic region, payer type, and race/ethnicity. Payer type was estimated for each person prescribed PrEP using a payer hierarchy of Medicaid, Medicare, commercial insurance, cash, and other payers. The number of PrEP users who received medication assistance program benefits from the manufacturer of PrEP also was estimated.

The annual number of PrEP users aged ≥16 years increased by 470%, from 13,748 in 2014 to 78,360 in 2016 (Table 1). In 2016, 65.0% of PrEP users were aged 25–44 years, and 0.1% were aged 16–17 years. Males accounted for 95.3% of all PrEP users. The percentage of PrEP users was highest in the Western U.S. Census Region (29.7%), followed by the Southern (27.2%) and Northeastern Regions (26.7%) and was lowest in the Midwestern Region (16.3%). Commercial health insurance was the payer for 81.0% of PrEP users’ medications and Medicaid for 12.2%. The number of PrEP users who received medication assistance program benefits from the manufacturer increased significantly, from 435 in 2014 to 5,437 in 2016.

**TABLE 1:** Annual number of persons aged ≥16 years prescribed HIV preexposure prophylaxis, by selected characteristics — IQVIA* Longitudinal Prescription Database, United States, 2014─2016

Characteristic	Year no (%)		
	2014	2015	2016
**Total**	**13,748 (100)**	**38,879 (100)**	**78,360 (100)**
**Sex**			
Male	12,624 (91.8)	36,845 (94.8)	74,639 (95.3)
Female	1,110 (8.1)	2,012 (5.2)	3,678 (4.7)
Unknown/Missing	14 (0.1)	22 (0.1)	43 (0.1)
**Age group (yrs)**			
16–17	22 (0.2)	29 (0.1)	64 (0.1)
18–24	953 (6.9)	3,223 (8.3)	7,382 (9.4)
25–34	4,687 (34.1)	14,766 (38.0)	30,959 (39.5)
35–44	3,825 (27.8)	10,156 (26.1)	19,989 (25.5)
45–54	2,845 (20.7)	7,564 (19.5)	13,913 (17.8)
55–64	1,080 (7.9)	2,543 (6.5)	5,046 (6.4)
≥65	336 (2.4)	598 (1.5)	1,007 (1.3)
**Census region**			
Northeast	3,411 (24.8)	10,110 (26.0)	20,909 (26.7)
Midwest	2,330 (17.0)	6,350 (16.3)	12,748 (16.3)
South	3,562 (25.9)	10,223 (26.3)	21,335 (27.2)
West	4,420 (32.2)	12,169 (31.3)	23,306 (29.7)
Other^†^	22 (0.2)	22 (0.1)	55 (0.1)
Unknown/Missing	3 (0.0)	5 (0.0)	7 (0.0)
**Payer type^§^**			
Medicaid/CHIP	1,430 (10.4)	4,547 (11.7)	9,542 (12.2)
Medicare	488 (3.6)	968 (2.5)	1,832 (2.3)
Commercial	9,980 (72.6)	31,993 (82.3)	63,430 (81.0)
Cash	163 (1.2)	262 (0.7)	732 (0.9)
Other^¶^	356 (2.6)	1,080 (2.8)	2,705 (3.5)
Unknown/Missing	1,331 (9.7)	29 (0.1)	119 (0.2)

**Abbreviation:** CHIP = Children's Health Insurance Program.

* https://www.iqvia.com/.

^†^ Other region included U.S. territories.

^§^ Payer type is a calculated hierarchical variable, thus numbers of each category are mutually exclusive. Before 2014, payer type information was not available for some of the specialty mail order suppliers.

^¶^ Other payer types included coupon/voucher programs, discount card programs, and federal or state assistance programs.

When length of TDF/FTC prescription drug use for PrEP was defined as >28 days rather than >30 days, the total number of PrEP users in 2016 increased 26%, from 78,360 to 98,599. Demographic and payer type distributions were similar using both algorithms (Table 2).

**TABLE 2:** Number of persons aged ≥16 years prescribed HIV preexposure prophylaxis based on different durations of drug use, by selected characteristics — IQVIA Longitudinal Prescription Database, United States, 2016

Characteristic	Length of drug use no. (%)	
	>30 days	>28 days
**Total**	**78,360 (100)**	**98,599 (100)**
**Sex**		
Male	74,639 (95.3)	92,042 (93.4)
Female	3,678 (4.7)	6,468 (6.6)
Unknown/Missing	43 (0.1)	89 (0.1)
**Age group (yrs)**		
16–17	64 (0.1)	175 (0.2)
18–24	7,382 (9.4)	10,984 (11.1)
25–34	30,959 (39.5)	39,243 (39.8)
35–44	19,989 (25.5)	24,177 (24.5)
45–54	13,913 (17.8)	16,646 (16.9)
55–64	5,046 (6.4)	6,067 (6.2)
≥65	1,007 (1.3)	1,307 (1.3)
Race/Ethnicity*		
White	22,574 (68.7)	26,832 (67.7)
Black	3,687 (11.2)	4,693 (11.8)
Hispanic	4,317 (13.1)	5,409 (13.6)
Asian	1,486 (4.5)	1,779 (4.5)
Unspecified	789 (2.4)	941 (2.4)
**Census region**		
Northeast	20,909 (26.7)	26,460 (26.8)
Midwest	12,748 (16.3)	15,704 (15.9)
South	21,335 (27.2)	27,119 (27.5)
West	23,306 (29.8)	29,217 (29.6)
Other	55 (0.1)	87 (0.1)
Unknown/Missing	7 (0.0)	12 (0.0)
**Payer type^†^**		
Medicaid/CHIP	9,542 (12.2)	12,732 (12.9)
Medicare	1,832 (2.3)	2,355 (2.4)
Commercial	63,430 (81.0)	76,767 (77.9)
Cash	732 (0.9)	2,332 (2.4)
Other^§^	2,705 (3.5)	4,206 (4.3)
Unknown/Missing	119 (0.2)	207 (0.2)

**Abbreviation:** CHIP = Children's Health Insurance Program.

* Percentages calculated among 32,853 (41.9%) >30-day users and 39,654 (40.2%) >28-day users with information on race/ethnicity available.

^†^ Payer type is a calculated hierarchical variable, thus numbers of each category are mutually exclusive. Persons who identified their race as white, black, Asian, or unspecified were all non-Hispanic. Persons who identified as Hispanic might be of any race.

^§^ Other payer type included coupon and voucher programs, discount card programs, and federal or state assistance programs.

Among the 78,360 PrEP users identified in 2016, information on race/ethnicity was available for 32,853 (41.9%), including 22,574 (68.7%) who were white, 3,687 (11.2%) who were black, 4,317 (13.1%) who were Hispanic, and 1,486 (4.5%) who were Asian. When stratified by sex, among the 1,146 female PrEP users with race/ethnicity data, 554 (48.3%) were white, 297 (25.9%) were black, and 201 (17.5%) were Hispanic (Figure).

**FIGURE:**
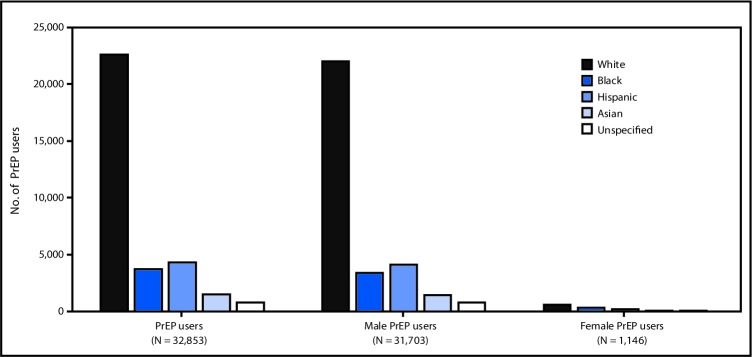
Number of PrEP users by sex and race/ethnicity*— IQVIA Longitudinal Prescription Database, United States, 2016 **Abbreviation:** PrEP = preexposure prophylaxis. * Among 32,853 (42%) persons with race/ethnicity data available, among all 78,360 PrEP users identified in 2016; information on sex was missing/unknown for four of these 32,853 persons.

## Discussion

Compared with recently published estimates based on an analysis of the MarketScan database with commercial health insurance billing claims, the estimated number of PrEP users was higher using this IQVIA database (*8*). This is because the IQVIA database contains all third party payers, including Medicaid, and prescriptions claims paid by medication assistance programs. The number of PrEP users with commercial insurance was similar in both analyses. In 2014, a total of 7,792 PrEP users with commercial insurance were identified in the MarketScan database, compared with 9,980 users with commercial insurance in the IQVIA database (*8*); in 2015, a total of 33,273 PrEP users with commercial insurance were identified in MarketScan,^†^ compared with 31,993 users with commercial insurance in IQVIA. The algorithm used in this study and in the MarketScan analysis defined postexposure prophylaxis as a TDF/FTC prescription for ≤30 days, resulting in a conservative estimate of PrEP use that might underestimate the number of PrEP users because persons might have been prescribed a 30-day supply of TDF/FTC for PrEP or postexposure prophylaxis. Persons prescribed TFD/FTC for ≤30 days might also have been using on-demand PrEP that is not taken daily. When a definition of postexposure prophylaxis as a TDF/FTC prescription for ≤28 days was used, the estimated number of PrEP users was higher. The true estimate of PrEP use likely falls between the estimate that defines PrEP use as a TDF/FTC prescription for >30 days and the one that defines it as >28 days. A validation study that compares estimates of PrEP use based on various algorithm definitions with a review of medical records will be helpful for future research.

Women accounted for 3,678 (4.7%) of the 78,360 PrEP users and 2.1% of the estimated 176,670 heterosexual women for whom PrEP is indicated (*6*). Among the estimated 1.1 million adults for whom PrEP is indicated, 303,230 (26.3%) were white, 500,340 (43.7%) were black, and 282,260 (24.7%) were Hispanic (*6*). However, among PrEP users with available race/ethnicity data in this study, 68.7% were white, 11.2% were black, and 13.1% were Hispanic. The large gap between the numbers of persons with indications for PrEP and those who were prescribed PrEP, and the low proportions of women and racial/ethnic minorities prescribed PrEP, suggests that more equitable implementation of PrEP recommendations for women and persons in racial/ethnic minority populations is needed. In addition, whereas men and women in the South had 52% of HIV diagnoses in the United States in 2016 (*8*), this study found that only 27% of the PrEP users were in the South.

The findings in this report are subject to at least four limitations. First, 58% of PrEP users identified in the IQVIA database did not have race/ethnicity information available. Race/ethnicity data were obtained from a convenience sample of a consumer database, in which persons who were older and had a credit history were more likely to be included. Although race/ethnicity data were not available for many PrEP users, this study suggests a substantial unmet prevention need for black and Hispanic populations who might benefit from PrEP. Second, PrEP users were identified using an algorithm that might be subject to misclassification bias. However, a similar algorithm was validated based on a review of electronic medical records (*10*). Third, the estimates were based on prescriptions dispensed rather than actual use. Finally, the IQVIA database did not include diagnosis data for 31% of persons, which might result in an overestimate of PrEP users by including persons potentially using TDF/FTC for treatment of HIV or hepatitis B infection. However, most persons (99%) with HIV in the IQVIA database had other antiretroviral medications in addition to TDF/FTC and were excluded.

Barriers to the provision of PrEP for persons in populations with the highest rates of annual HIV diagnoses, such as black and Hispanic men and women, need to be better understood to help guide the development of interventions to increase access to and utilization of PrEP. Focused public health efforts to support increasing PrEP prescriptions for persons in populations who might benefit from its use could increase the impact of PrEP on HIV incidence in the United States.

**Summary**

**What is already known about this topic?**

In 2015, approximately 1.1 million adults were at risk for acquiring human immunodeficiency virus infection and had indications for preexposure prophylaxis (PrEP); 26.3%, 43.7%, and 24.7% were white, black, and Hispanic, respectively.

**What is added by this report?**

In 2016, among 78,360 persons who filled prescriptions for PrEP in the United States, women accounted for only 4.7%. Among PrEP users with available race/ethnicity data, 68.7%, 11.2%, 13.1%, and 4.5% were white, black, Hispanic, and Asian, respectively.

**What are the implications for public health practice?**

The gap between numbers of persons with PrEP indications and those prescribed PrEP was substantial, especially among persons in female, black, and Hispanic populations. Focused efforts are needed to increase the impact of PrEP in the United States.

## Footnotes

*
https://www.iqvia.com/locations/united-states/commercial-operations/essential-information/prescription-information .

† MarketScan data came from Table 11, Monitoring selected national HIV prevention and care objectives by using HIV surveillance data—United States and 6 dependent areas, 2015. HIV Surveillance Supplemental Report 2017; vol. 22, no. 2. https://www.cdc.gov/hiv/pdf/library/reports/surveillance/cdc-hiv-surveillance-supplemental-report-vol-22-2.pdf.
